# Hydrogenation of Ethyl Acetate to Ethanol over Ni-Based Catalysts Obtained from Ni/Al Hydrotalcite-Like Compounds 

**DOI:** 10.3390/molecules15085139

**Published:** 2010-07-29

**Authors:** Beixiao Zhang, Lu Lin, Junping Zhuang, Ying Liu, Lincai Peng, Longfei Jiang

**Affiliations:** State Key Laboratory of Pulp and Paper Engineering, South China University of Technology, Guangzhou 510640, Guangdong, China

**Keywords:** hydrogenation, ethyl acetate, ethanol, Ni/Al hydrotalcite-like compounds

## Abstract

A series of Ni-based catalysts were prepared using hydrogen reduction of Ni/Al hydrotalcite-like compounds (Ni/Al HTlcs) synthesized by coprecipitation. The physico-chemical properties of Ni/Al hydrotalcite-like compounds and the corresponding Ni-based catalysts were characterized using inductively coupled plasma (ICP), BET surface areas, X-ray diffraction (XRD), Fourier transform infrared (FTIR) spectroscopy and scanning electron microscopy (SEM) techniques. The results indicated that Ni/Al HTlcs with layered structures could be successfully prepared by the coprecipitation method, and the characteristic HTlcs reflections were also observed in the XRD analysis. The NiO and Ni^0 ^phases were identified in all Ni-based catalysts, which displayed randomly interconnected pores and no layer structures. In addition, the studies also found the Ni/Al HTlcs and Ni-based catalysts had high specific surface areas, low pore volumes and low pore diameters. The catalytic hydrogenation of ethyl acetate to ethanol with Ni-based catalysts was also investigated. Among the studied catalysts, RE1NASH-110-3 showed the highest selectivity and yield of ethyl acetate to ethanol, which were 68.2% and 61.7%, respectively. At the same time, a major by-product, butyl acetate, was formed due to an ester-exchange reaction. A proposed hydrogenation pathway for ethyl acetate over Ni-based catalysts was suggested.

## 1. Introduction

**Scheme 1:**
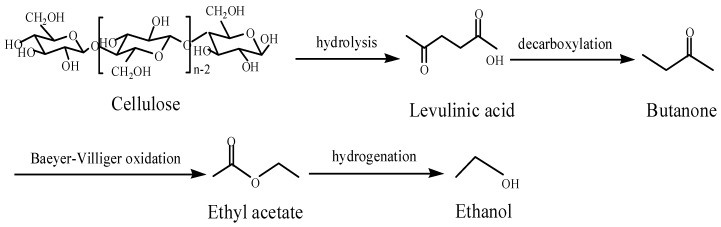
The pathway of conversion of lignocellulose to ethanol via levulinic acid.

Exploitation of diverse methods to produce fuel ethanol from lignocellulose is meaningful for the research and development activities in the alternative energy field. Ethyl acetate can be produced from lignocellulosic biomass via transformation of glucose to levulinic acid. There are two steps in the transformation of levulinic acid into ethyl acetate [1,2], one is the decarboxylation of levulinic acid to butanone [3], the other is Baeyer-Villiger oxidation of butanone to ethyl acetate [4]. Ethyl acetate can then be further reduced to ethanol (Scheme 1). As one kind of potential vehicle fuel, fuel ethanol is useful to reduce the consumption of gasoline all over the world, and will provide much more sustainable options for the production of biomass fuels. 

Reduction of esters to the corresponding alcohols is one of the fundamental reactions in organic chemistry, and is employed in a large number of chemical processes. In general, there are two main types of processes to achieve such a reduction. One is through traditional hydride processes, in which silyl or metal hydride salts, such as LiAlH4 and NaBH4 are used, the other is through hydrogenation processes, where molecular hydrogen is employed. From a practical point of view, hydrogenation processes can be run with small amounts of catalyst and in the presence of small quantities or even in the absence of solvent. Furthermore, hydrogenation processes do not require highly reactive and expensive hydrides, engender less aqueous waste, and are environmentally friendly and economically attractive [5].

Hydrogenation of esters to the corresponding alcohols is usually carried out according to the stoichiometry shown in Equation 1:

R_1_COOR_2 _+ 2H_2_= R_1_CH_2_OH+ R_2_OH (1)

Various catalysts have been successfully used in these hydrogenation reactions, including both homogeneous and heterogeneous catalysts. Homogeneous catalytic hydrogenation of esters has been rarely explored and is mostly limited to activated esters [6,7,8]. Roger and coworkers reported that the anionic ruthenium hydride complex K_2_[(PPh_3_)_3_(PPh_2_)Ru_2_H_4_]·(C_6_H_14_O_3_)_2_ could catalyze the hydrogenation of activated esters, while ethyl acetate was hydrogenated to ethanol with only 8% yield and aromatic esters could not be hydrogenated [7]. By contrast, heterogeneous catalysts are widely used in hydrogenation of esters, for example, copper-based catalysts exhibit high activity and selectivity for hydrogenation of esters to alcohols [9], but the use of the catalysts requires severe reaction conditions at 250–350 °C and approximately 10-20 MPa. Some catalysts based on transition metals such as Sn, Ru, Pd, Pt, Re and Rh are also reported to be usable in the reduction of esters [10]. Abdellah et al. claimed that the hydrogenation of ethyl acetate to ethanol could be carried out with various Rh/Sn/SiO_2_ catalysts, the highest yield was 68% [11]. Okumura *et al*. described that Rh/one-atomic-layer GeO_2_/SiO_2_ catalysts had exhibited high activity and selectivity for ethyl acetate hydrogenation [12].

Hydrotalcite belongs to the large class of anionic clays with layered brucite structures and a general formula of Mg_6_Al_2_(OH)_16_CO_3_·4H_2_O. Hydrotalcite-like compounds (HTlcs), also known as layered double hydroxides, consist of brucite-like layers with positive charge and anionic compounds in the interlayer, their general formula is [M(II)_1-__x_M(III)_x_(OH)_2_]^x+^(A^n-^_x/n_)·mH_2_O, where M(II) may be Mg, Zn, Co, Cu, Fe, Ni and Mn, M(III) may be Al, Cr, Fe and V, and A is usually CO_3_^2-^ anions [13]. A wide variety of HTlcs have been synthesized by modification of the lamellar and interlamellar regions.

Due to its large surface area, basic properties, high metal dispersion and stability against sintering, hydrotalcite-like compounds and their derived forms have recently been attracting considerable attention for their potential applications in oxidation [14], hydrogenation [15,16,17], steam reforming [18], dehydrogenation [19], condensation [20], alkylation [21], Meerwein–Ponndorf–Verley reactions [22] and so on. Chen *et al*. investigated the hydrogenation of methyl benzoate to benzaldehyde on manganese oxide catalysts prepared from Mg/Mn/Al hydrotalcite-like compounds [15], in which the conversion of methyl benzoate and selectivity to benzaldehyde with a dosage of 3% K/Mg_0.2_Mn_1.8_Al_1_ catalyst at 390 °C were 90.0% and 88.0%, respectively. Boyapati *et al*. reported that the hydrogenation of aromatic and heterocyclic aldehydes at atmospheric pressure by calcinated Ni/Al hydrotalcite generated high yields of the reduced products, and the catalyst could be reused for several cycles with consistent catalytic activity and selectivity [16]. 

It is well known that nickel is a cheaper metal compared with the noble metals such as palladium, rhodium and ruthenium, and it is widely used for hydrogenation of organic compounds. In the present study, Ni/Al HTlcs were synthesized by a coprecipitation method. The physicochemical properties of Ni/Al hydrotalcite-like compounds and the corresponding Ni-based catalysts were characterized using various techniques. The Ni-based catalysts were used for the hydrogenation of ethyl acetate to ethanol. The proposed hydrogenation pathway for ethyl acetate over Ni-based catalysts was also studied. 

## 2. Results and Discussion

### 2.1. Sample characterization

The Ni/Al molar ratios of the prepared hydrotalcite-like compounds are shown in Table 1. The results showed that the actual Ni/Al molar ratio was almost identical to the experimental design value, which indicated that the precipitation process was efficient, the Ni/Al hydrotalcite-like compounds with different Ni/Al molar ratio could be successfully prepared by coprecipitation methods.

The specific surface areas (S_BET_), pore volume (Vp) and average pore diameters (dp) of various samples were studied, as shown in Table 2. The results indicated that the specific surface area and pore volume of Ni/Al HTlcs increased as the Ni/Al molar ratio increased, which could also be seen in the resultant reduced products, whereas average pore diameters decreased with the increase of Ni/Al molar ratio whether the Ni/Al HTlcs were prepared by reflux at 80 °C or maintained under static conditions. When the Ni/Al molar ratio varied from 1:1 to 5:1, the specific surface areas of Ni-based catalysts with reflux treatment increased from 100.6 to 139.1 m^2^/g, and ranged from 105.7 to 149.6 m^2^/g, respectively, under static conditions. The Vp values drifted from 0.24 to 0.40 mL/g and increased with the increase of specific surface areas. However, the average pore diameters decreased conversely in the range from 18.16 to 10.25 nm. The nucleation and growth of HTlcs was a complicated process, and the crystal formation was attributed to the influence of several factors. The ionic radius of nickel is larger than that of aluminum, so the incorporation of more nickel could lead to lattice expansion [23], which might be one of the reasons for the observed changes of S_BET_, Vp and dp as the Ni/Al molar ratio increased. In addition, crystallization conditions and reduction temperatures in the preparation could also influence the pore structure.

**Table 1:** The molar ratio of Ni/Al hydrotalcite-like compounds.

Catalyst	Ni/Al ratio design	Actual Ni/Al ratio
1NAR-80-20	1	1.17
3NAR-80-20	3	3.21
5NAR-80-20	5	5.32
1NASH-110-3	1	1.22
3NASH-110-3	3	3.18
5NASH-110-3	5	5.27

**Table 2:** Specific surface area and pore structure analysis of the Ni/Al HTlcs and Ni-based catalysts.

Catalyst	S_BET_(m^2^/g)^a^	V_p_(mL/g)^b^	*d*p(Å)^c^
1NASH-110-3	124.6	0.32	16.07
5NASH-110-3	169.3	0.43	9.35
RE1NAR-80-20	100.6	0.24	18.16
RE3NAR-80-20	106.5	0.29	17.92
RE5NAR-80-20	139.1	0.37	13.89
RE1NASH-110-3	105.7	0.26	17.75
RE3NASH-110-3	116.2	0.30	16.23
RE5NASH-110-3	149.6	0.40	10.25

a. Specific surface area, b. Pore volume, c. Average pore diameter

**Figure 1:**
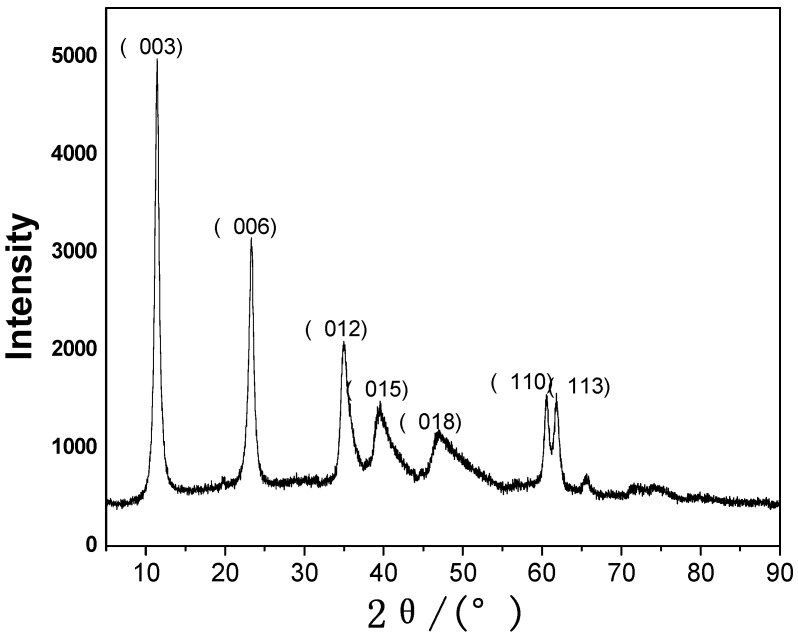
XRD patterns of Ni/Al HTlcs.

The X-ray diffraction patterns of the fresh Ni/Al HTlcs are shown in Figure 1. Sharp and symmetric reflections for the basal (003), (006), (110) and (113), and less symmetric peaks in the (012), (015) and (018) planes were the characteristic reflections of HTlcs. The results indicated that HTlcs with layered structures were the main component in the samples, and no signals of other crystalline phases were detected, which agreed with the literature reports [13,25,26]. Therefore, it can be concluded that HTlcs with single crystal phase were successfully prepared by coprecipitation method used. 

**Figure 2:**
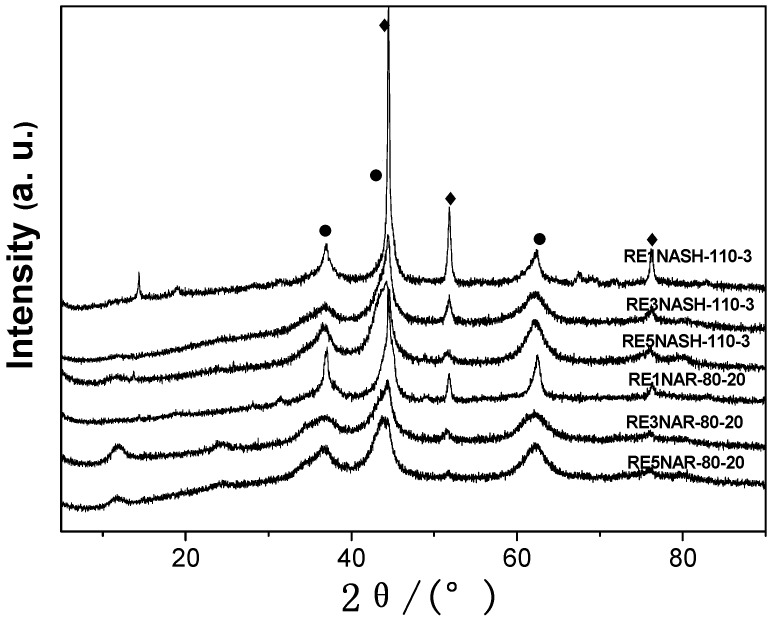
XRD patterns of Ni-based catalysts obtained from Ni/Al HTlcs: ●-NiO, ♦-Ni.

As shown in Figure 2, the characteristic reflections of HTlcs disappeared after reduction, but the typical reflections of NiO and Ni were all detected by XRD. When Ni/Al HTlcs were reduced at 300 °C, the hydrogen promoted the decomposition of carbonate anions to CO_2 _and of NO_3_^-^ to NO, so structural holes were generated on the surface of Ni/Al HTlcs, and these structural holes favored the accessibility to the NiO particles [24]. The formation of a new crystal metallic Ni phase was attributed to the reduction of NiO to Ni^0^ with the release of water after catalytic precursors were reduced at 300 °C [25]. The NiO and Ni features of the reduced HTlcs under stirring reflux were affected by the Ni/Al molar ratio, the X-ray patterns of NiO became broad and asymmetric as the Ni/Al ratio increased. 

Nevertheless, the Ni X-ray patterns became sharp and intense as the Ni/Al ratio decreased. On the other hand, the low relative intensity of the XRD signals suggested a high metal dispersion after the reduction treatment [33]. These changes were also observed obviously in the reduced Ni/Al HTlcs with static hydrothermal treatment. In conclusion, the lamellar structure mostly collapsed with the decomposition of CO_3_^2-^ and NO_3_^-^ and the release of partial H_2_O in the interlayer, which could be proven by the disappearance of the characteristic reflections of HTlcs.

**Figure 3:**
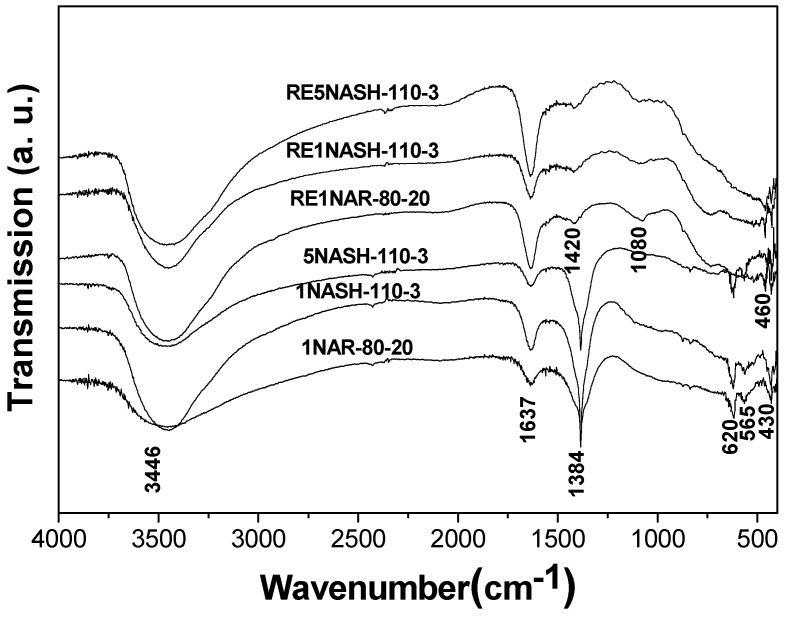
FTIR spectra of Ni/Al HTlcs and Ni-based catalysts.

The FTIR analysis of different HTlcs and reduced HTlcs is shown in Figure 3. 1NAR-80-20, 1NASH-110-3 and 5NASH-110-3 displayed similar spectra showing typical hydrotalcite-like compounds peaks and three general types of IR-active vibrations could be distinguished, which were the molecular vibrations of hydroxyl groups, lattice vibrations of the octahedral layers and vibrations of the interlayer anions [27]. The intense and broad band observed about 3,460 cm^-1^ was assigned to the OH stretching of water molecules and hydroxyl groups of the brucitic layers. The absorption at 1,637 cm^-1^ was attributed to *δ*_H2O_ vibration of interlayer water molecules. Moreover, the presence of the CO_3_^2-^ interlayer anions was manifested by the ν_3_ mode around 1,360 cm^-1^. Evidence for the presence of NO_3_^-^ was also found with the characteristic vibrations of ν_3_ at 1,385 cm^-1^. Bands below 1,000 cm^-1^ were due to stretching and deformation modes of M-O, M-O-M, and O-M-O moieties in the brucite-like layers [28]. The peak at 620 cm^-1^ might be related to the presence of Ni cations and Ni–OH translation, the peak at 565 cm^-1^ was due to the Al-OH translational mode, while the sharp and intense band at 430 cm^‑1^ could be assigned to the condensed [AlO_6_]^3-^ group or a single Al–O bond [29].

The spectra of the samples reduced at 300 °C showed that the bands at 3,460 and 1,637 cm^-1 ^did not obviously change. However, no bands corresponding to the interlayer nitrate anions were observed, the band of CO_3_^2- ^groups at 1,360 cm^-1 ^also disappeared, which might be caused by the decomposition of NO_3_^-^ anions to NO and partial conversion of CO_3_^2-^ anions to CO_2_, respectively. The results indicated that the lamellar structure mostly collapsed, as also testified by the XRD patterns of the reduced Ni/Al HTlcs. The absorption bands between 850 and 1,500 cm^-1^ were consistent with that of the calcinated HTlcs, and two new bands at 1,420 and 1,080 cm^-1^ were found and assigned to the ν_2_ and ν_3_ modes of the CO_3_^2-^ anions [27]. Moreover, the bands of NiO were also observed at 460 cm^-1^ [30]. 

**Figure 4:**
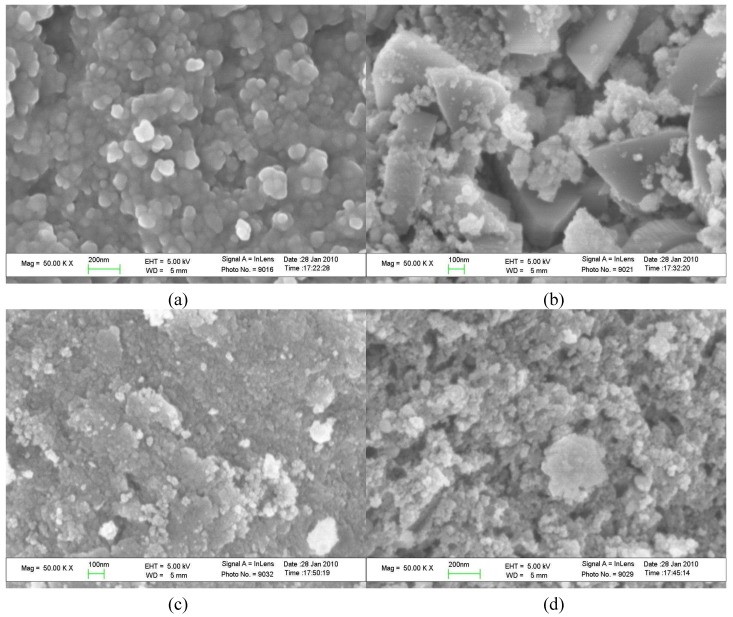
SEM photographs of Ni-based catalysts: (a) RE1NAR-80-20; (b) RE1NASH-110-3; (c) RE5NAR-80-20; (d) RE5NASH-110-3.

SEM morphology of the samples RE1NAR-80-20, RE1NASH-110-3, RE5NAR-80-20 and RE5N ASH-110-3 is shown in Figure 4. The results showed that the lamellar structure of HTlcs was destroyed and microporous structure of Ni-based catalysts was formed. When the Ni/Al molar ratio increased from 1:1 to 5:1, the particle size of samples decreased conversely. The reduced sample of Ni/Al molar ration 5:1 had a large specific surface area, which was also confirmed by specific surface area analysis. Among the four samples, RE1NAR-80-20 exhibited homogeneous spherical structure, the morphology RE1NASH-110-3 of was granular or triangular, whereas RE5NAR-80-20 appeared agglomerated and RE5NASH-110-3 presented a honeycombed morphological structure. From these results, it could be concluded that Ni-based catalysts had randomly interconnected micropores, which was in accordance with the results reported in the literature [23]. The morphological changes of the resultant samples might be due to different Ni/Al molar ratio and variation of crystallization conditions. 

### 2.2. Catalytic hydrogenation of ethyl acetate to ethanol

The activity and selectivity of catalytic hydrogenation of ethyl acetate to ethanol on Ni-based catalysts are shown in Table 3. According to the hydrogenation reaction stoichiometry, one molecule of ethyl acetate would be reduced to two molecules of ethanol. However, hydrogenation reaction by-products such as butyl acetate, acetaldehyde, ethyl ether, methane and ethane were also detected. Among these by-products, butyl acetate might be produced by ester-exchange reaction between the original ester and the alcohol used as solvent, a phenomenon also reported in the literature [10]. In all Ni-based catalysts from Ni/Al hydrotalcite-like compounds, the conversion of ethyl acetate was similar and around 90%, but the different catalysts showed different selectivity for ethanol. Nickel as a base metal catalyst could offer much promise for the hydrogenation of organic compounds including ester reduction [31]. Interestingly catalysts with high proportions of Ni didn’t stand for their high hydrogenation activity. Monzon *et al*. reported that the conversion rate, selectivity and yield for the hydrogenation of acetylene to ethylene decreased as the Ni concentration increased [28]. This phenomenon was also seen in our experiments. 

**Table 3:** Hydrogenation of ethyl acetate on Ni-based catalysts.

Catalyst	Conversion	Selectivity			
		Ethanol	Butyl acetate	Ethyl ether	Acetaldehyde
RE1NAR-80-20	90.6	64.6	19.3	0.4	6.2
RE3NAR-80-20	91.6	61.5	21.5	0.3	8.6
RE5NAR-80-20	90.4	59.1.	23.7	0.3	8.9
RE1NASH-110-3	90.5	68.2	14.5	0.7	4.3
RE3NASH-110-3	90.3	64.3	17.3	0.5	6.8
RE5NASH-110-3	89.2	62.5	18.9	0.4	8.5
Nickel powder	76.3	70.3	14.5	0.6	3.8

*Reaction conditions*: catalyst dosage 0.14 g, temperature 250 °C, time 9 h, hydrogen pressure 6 MPa (at 25 °C), ethyl acetate 0.02 mol.

Results from the experiments also indicated that the selectivity for ethanol fell with the rise in Ni/Al mole ratio, whereas the selectivity for butyl acetate and acetaldehyde rose conversely. The Ni-based catalyst RE1NASH-110-3 showed the highest selectivity and yield of ethyl acetate to ethanol, which were 68.2% and 61.7%, respectively. The results were lower than the results of 99% selectivity and 79 wt% yield of ethanol reported by Bournonville *et al*. with Raney nickel and tetrabutyltin, but were higher than the results of 49.5% conversion and 46.5 wt% yield of ethanol with 2.5%Ni/3%Sn/SiO_2 _[32]. The conversion of ethyl acetate and yield of ethanol with pure nickel powder were 76.3% and 53.6%, respectively, ranking as the lowest yield for ethanol among the Ni-based catalysts examined in these experiments. In conclusion, RE1NASH-110-3 is an efficient catalyst for the catalytic hydrogenation of ethyl acetate to ethanol.

### 2.3. Reaction pathway for the hydrogenation of ethyl acetate to ethanol

**Scheme 2:**
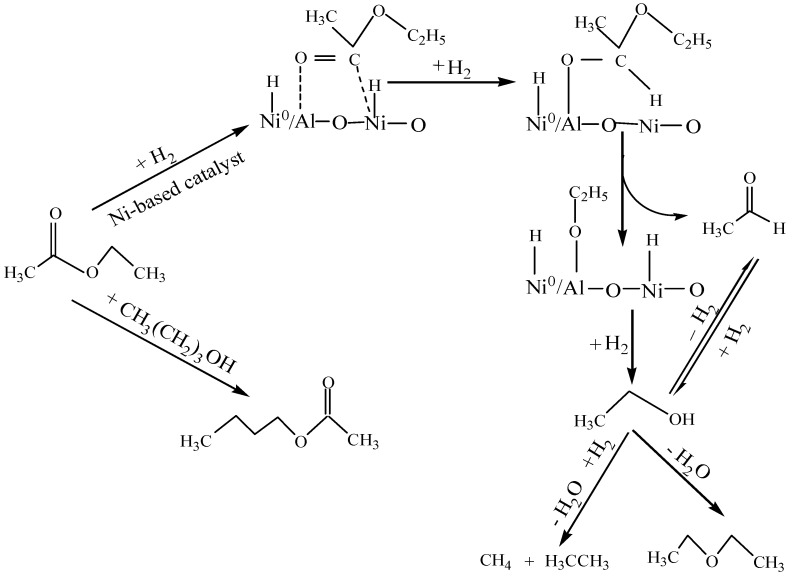
The proposed reaction pathway for hydrogenation of ethyl acetate.

According to the experimental results and the related literatures, a plausible reaction pathway for hydrogenation of ethyl acetate to ethanol is proposed as Scheme 2. In the pathway, ethyl acetate is firstly absorbed on the active Ni^2+^ cation sites in association with aluminium oxide through a relatively weak acyl structure [33], the metal active sites in the catalysts are responsible for the carbonyl activation, which also seems to be the reason for the increased conversion. The weak acyl structure facilitates desorption of the resultant products from the surface of the catalysts. Alumina without active centers is a better support for dispersing nickel crystallites (Ni^0^). Ni^0 ^as another active sites is very active for adsorbing dissociative H_2_ and may supply Ni^2+^ cations with activated atomic hydrogen to obtain nickel hydride, and the transfer of the hydrogen atom in nickel hydride to the carbonyl carbon atom forms hemiacetal groups according to the Adkins model [34]. Subsequently, acetaldehyde and an adsorbed ethoxy group are obtained through the cleavage of the C-O bond of the hemiacetal group, and then the ethoxy group would be quickly hydrogenated to ethanol. In addition, side reactions of ethanol also happen, for example, reversible dehydrogenation of ethanol to aldehydes, hydrogenation and dehydration of ethanol to methane and ethane, inter-molecular dehydration of ethanol to ethyl ether, ester-exchange reaction between the original ester and the solvent alcohol to butyl acetate. Hence, hydrogenation of ethyl acetate over Ni-based catalysts not only leads to the formation of ethanol but also the formation of a variety of by-products such as acetaldehyde, methane, ethane, ethyl ether and butyl acetate. How to control the adverse side-reactions and increase the yield of ethanol is the subject of further studies. 

## 3. Experimental

### 3.1. Materials

Nickel powder, ethanol and butyl acetate (all >99% purity) were purchased from Aladdin Chemicals. Acetaldehyde and ethyl ether purchased from BASF Chemical Company were analytical grade and filtered before use. Other reagents and chemicals were all of analytical grade, obtained from Sinopharm Chemical Reagent Co. Ltd and used directly without any further purification.

### 3.2. Preparation of Ni/Al HTlcs

Six samples of Ni/Al hydrotalcite-like compounds under two different crystallization conditions were prepared by a coprecipitation reaction at constant pH value. A mixture of solutions of Ni (NO_3_)_2_· 6H_2_O and Al (NO_3_)_3_·9H_2_O with Ni/Al molar ratios of 1, 3 and 5, respectively, was added along with 1M of NaOH and Na_2_CO_3_ solutions to a beaker containing deionized water (approximately 100 mL) at a constant pH of 8.5 ± 0.2. The solutions were added dropwise under vigorous stirring at room temperature over about 4 h. The resultant suspension was stirred continuously under reflux for 20 h at 80 °C or maintained under static conditions (static hydrothermal treatment) for 3 h at 110 °C in a stainless steel autoclave, respectively. The products were cooled to room temperature, filtered and washed thoroughly with deionized water, then dried for 16 h at 100 °C. The samples were reduced in a stainless steel autoclave at 300 °C for 4 hours, at 4 MPa of hydrogen pressure and with a stirring speed of 600 rpm. In the end, the Ni^2+^ in the samples was thus reduced to Ni^0^, as confirmed by the color change from the initial green color to gray after the reaction.

The resultant samples were named as follows: MNAR-T-t and MNASH-T-t for the hydrotalcite-like compounds samples under stirring reflux and static hydrothermal treatment, respectively. M of 1, 3 and 5 stand for the series with Ni/Al molar ratio of 1/1, 3/1 and 5/1, respectively. T is the heating temperature (80 or 110 °C), and t is the crystallization time (3 or 20 h). REMNAR-T-t and REMNASH-T-t are the reduced HTlcs.

### 3.3. Characterization of catalysts

The metal contents of the catalysts were determined with an inductively coupled plasma-atomic emission spectrometer (IRIS 1000, Thermo Jarrell Ash) under standard conditions. Ni/Al hydrotalcite-like compounds were digested with a mixture of concentrated HNO_3_ and HClO_4_, and diluted with 2% of HNO_3_.

X-ray diffraction (XRD) analysis was performed on a Rigaku powder diffractometer (Rigaku, Japan) with Cu K_α_ radiation at 40 kV and 40 mA. Scans were performed over the 2θ ranges from 3° to 90° at a step of 0.02° and a count time of 17.7 s at each step. The crystalline phases were identified by Powder Diffraction Standards (JCPDS) files.

Fourier transform infrared (FTIR) spectroscopy of Ni/Al HTlcs and their reduced products were recorded over the range from 400 to 4,000 cm^-1^ on a SpectrumGX FT-IR spectrometer. The powder samples were mixed with KBr and pressed into thin wafers before being measured.

BET surface areas were measured from the nitrogen adsorption isotherms at 77 K using a Micromeritics TriStar II instrument, Samples were degassed under vacuum at 120 °C for 6 h with a Micromeritics VacPrep 061 sample degassing system. The same equipment automatically calculated pore radii and pore volumes by the methods of Barret-Joyner-Halenda (BJH).

Scanning electron micrographs of Ni-based catalysts were taken with an LEO1530VP Field Emission Electronic Microscope at acceleration voltages of 5 kV, with a working distance of 5 mm and magnification values up to 100,000×.

### 3.4. Catalytic hydrogenation of ethyl acetate

Hydrogenation of ethyl acetate was performed in a 200 mL stainless steel autoclave. Ethyl acetate (0.02 mol) in *n*-butanol (50 mL) was introduced into the autoclave, and then samples of Ni/Al hydrotalcite-like compounds or nickel powder (0.14 g) was added in different experimental designs. After purging the autoclave five times with hydrogen, the autoclave was pressurized with hydrogen to 6.0 MPa. The reaction temperature was fixed at 250 °C for 9 h. The stirring rate was adjusted to 600 rpm. The temperature was decreased rapidly to room temperature with a condenser after the reaction.

Qualitative analysis of the resultant products was performed by gas chromatograph-mass spectrometry (HP 6890-5975 GC-MS with a 5972 mass selective detector). A HP-5 5% phenylmethyl siloxane column (30 m × 0.32 mm × 0.25 μm) was used. The injector temperature was 250 °C, the detector temperature was 270 °C with a helium flow rate of 0.8 mL/min. Keeping an initial oven temperature at 35 °C for 6 min, and then improving the oven temperature to 110 °C at a rate of 15 °C/min, to 250 °C with a rate of 20 °C/min and then keeping at 250 °C for 3 min. The mass spectrometer was used in electron impact (EI) ionization mode under 70 eV of electron energy. Methane and ethane was collected and qualitatively analyzed by GC-MS.

Quantitative analysis of the products was performed using an Agilent 6890 gas chromatograph with a ﬂame ionisation detector (FID). A HP-5 5% phenylmethyl siloxane column (30 m × 0.32 mm × 0.25 μm) was used. Nitrogen at a flow rate of 0.8 mL/min was used as a carrier gas. The injector temperature, detector temperature and temperature program of oven were similar to the qualitative analysis.

## 4. Conclusions

Six hydrotalcite-like compounds with different Ni/Al molar ratios had been successfully synthesized under two different crystallization conditions, the Ni/Al HTlcs and their reduced products were characterized by ICP, BET, XRD, FITR and SEM. With the increase of Ni/Al molar ratio, an increase in BET areas, pore volume and the diminution of pore diameter were observed in Ni/Al HTlcs and their reduced products. NiO particles and nickel crystallites were obtained after reduction of hydrotalcite-like compounds. Partial dehydration and decomposition of NO_3_^-^ to NO and CO_3_^2-^ anions to CO_2_ also took place, which led to the collapse of the lamellar structure. 

Ni-based catalysts obtained from the reduction of hydrotalcite-like compounds were found to be efficient in the reduction of ethyl acetate under different hydrogenation conditions. Among these catalysts, RE1NASH-110-3 showed the highest selectivity (68.2%) and yield (61.7%) for the conversion of ethyl acetate to ethanol. Ethanol was formed during the process of conversion via hemiacetal and ethoxy group intermediates. In addition, by-products such as aldehydes, ethyl ether, methane, ethane and butyl acetate were also detected in the reaction process.
